# Comparison of the Efficacy of Combination Therapy of Prednisolone - Acyclovir with Prednisolone Alone in Bell’s Palsy

**Published:** 2015

**Authors:** Ali KHAJEH, Afshin FAYYAZI, Gholamreza SOLEIMANI, Ghasem MIRI-ALIABAD, Sara SHAYKH VEISI, Behrouz KHAJEH

**Affiliations:** 1 Department of Pediatrics, Children and Adolescent Health Research Center, Zahedan University of Medical Sciences, Zahedan, Iran.; 2 Department of Pediatrics, Hamedan University of Medical Sciences, Hamedan, Iran.; 3 General Physician, Zahedan University of Medical Sciences, Zahedan, Iran.; 4 Student of Pharmacy , Zabol University of Medical Science , Zabol, Iran

**Keywords:** Bell’s palsy, Prednisolone, Acyclovir, Children

## Abstract

**Objective:**

Bell’s palsy is a rapid onset, usually, unilateral paralysis of the facial nerve that causes significant changes in an individual’s life such as a decline in personal, social, and educational performance. This study compared efficacy of combined prednisolone and acyclovir therapy with prednisolone alone.

**Materials & Methods:**

This study is a randomized controlled trial conducted on 43 Children (2–18 years old) with Bell’s palsy. The first group of 23 patients was treated with prednisolone and the remaining patients were treated with a combination of prednisolone and acyclovir. The required data were extracted, using an informational form based on the House-Brackmann Scale, which grades facial nerve paralysis. The data were analyzed with Mann-Whitney test using SPSS version 16.

**Results:**

The mean age of the first and second group were 8.65 ± 5.07 and 8.35 ± 4.92 years, respectively, (p=0.84). Sixty one percent and 39% of patients in the first group, and 45% and 55% of patients in the second group were male and female, respectively. No significant differences exist between the groups in terms of age and gender. The rate of complete recovery was 65.2% in group I and 90% in the group II (p=0.04).

**Conclusion:**

The results of this study showed that the combined prednisolone and acyclovir therapy of patients with Bell’s palsy is far more effective than treatment with prednisolone alone. Actually, age and gender had no impact on the rate of recovery.

## Introduction

Introduction Bell’s palsy is a rapid onset, idiopathic facial nerve paralysis with an incidence rate of 20 per 100,000 per year (1). Facial paralysis can range from mild to complete paralysis and can improve within a year (2). The incidence rate is equal in both genders, occurs at any age, and both sides may be affected equally (3). Bell’s palsy is uncommon in children less than two years of age and a thorough examination should be performed to find out the reason for it (2). Despite uncertainty of the actual mechanisms of the disease and it may arise due to inflammation along the labyrinth of facial nerve bony canal that results in compression and demyelination of axon and impairment of nerve blood flow (4). Genetic factors, vascular ischemia, inflammatory factors secondary to viral infections, and autoimmune disorders are proposed as the underlying causes of Bell’s palsy. However, its cause is still unknown (5). Many viruses, including HIV, EBV, and HBV are suspected as triggering organisms, but HSV is the most involved (6). The main clinical symptom of Bell’s palsy is facial motor dysfunction (5). Patients complain typically about weakness or complete paralysis of all muscles on one side of the face. A lack of complete recovery of facial symmetry in the long term potentially affects the quality of life such as difficulty in drinking, eating, talking, and psychosocial problems, and creates a remarkable disruption in social activities (2, 7). The disease prognosis is good and almost 70% of patients recover completely within 6 months of treatment, though 30% of patients get residual symptoms, such as paresis, contracture, and face spasms or synkinesis (7). Treatment of these patients is controversial and variable (8). Prednisone and acyclovir are widely used separately or in combination; however, their effectiveness has been weakly evidenced (9, 10). Although there is no treatment of choice for this disease, corticosteroids are widely prescribed as the initial treatment of Bell’s palsy to reduce swelling and inflammation of the facial nerve (1, 5). The effects of antiviral therapies in Bell’s palsy are not confirmed so far and the question whether adding antiviral therapy to other treatments, such as corticosteroid therapy, can improve the disease better and faster than corticosteroids alone (1). Nevertheless, given the possible association of Bell’s palsy with viral infections such as HSV, antiviral therapy seems logical (5, 11). This study compares the efficiency and efficacy of acyclovir, prednisolone separately; and in combination in the treatment of Bell’s palsy in children.

## Materials & Methods

This study was performed as a randomized controlled trial (RCT). Forty-three patients with acute unilateral peripheral facial palsy with an age range of 2–18 years were enrolled in the study. Exclusion criteria included patients with paralysis of other cranial nerves, passing more than 3 days of symptoms onset, patients with less than 2 years of age and older than 18 years, presence of secondary causes of the 7th nerve palsy, suspicion of meningitis, vasculopathy, Ramsey Hunt syndrome, peptic ulcer, anti-herpetic treatment within the last 2 weeks, and sensitivity to acyclovir. The first group received 2 mg/ kg/day prednisolone, and the second group was treated with a combination of 2 mg/kg/day prednisolone and 10 mg/kg acyclovir every 8 hours for 7 days. In suspected cases, patients were examined to rule out other causes of the 7th nerve palsy, and imaging studies revealed a CP angle tumor in a patient with Bell’s palsy who was then excluded from the study. Informed consent was obtained from parents of children. This study was approved by the ethics committee of the University. The required data were extracted using an informational form based on the House-Brackmann Scale (12), which grades facial nerve paralysis. The patients were reassessed in terms of recovery rate at the end of the first and third months of treatment. Based on the HouseBrackmann criteria, the response to treatment are graded as complete recovery (grade 1), partial recovery (grade 2–5), and no response (grade 6). The data were analyzed using SPSS (ver 16) through comparing the efficacy of the two treatment regimens with Mann-Whitney and Fisher’s exact tests. P-value less than 0.05 were considered significant.

## Results

Table 1 depicts the demographic characteristics of patients. Among the 43 patients with Bell’s palsy in this study, 23 patients were treated with prednisolone and 20 patients with combination of prednisolone and acyclovir. There were no noteworthy differences in case of age and gender between the two groups (p > 0.05).

**Table 1:** Frequency of Bell’s palsy Patients according to the Age and Gender in Two Groups

Group		Prednisolone group	Combined Group	P-Value
Gender	Male	14(61 %)	9(45%)	0.29
	Female	9(39% )	11(55% )	
Mean age (yr)		8.65±5.07	8.35±4.92	0.84

Table 2 shows the treatment results. According to this, it can be concluded that the rate of recovery in patients Comparison of the Efficacy of Combination Therapy of Prednisolone - Acyclovir with Prednisolone Alone in Bell’s Palsy Iran J Child Neurol. 2015 Spring Vol 9 No 2 19 with Bell’s palsy treated with combined prednisolone and acyclovir was much more than that of prednisolone therapy alone (p = 0.04) and had lower sequels after treatment.

**Table 2:** Comparison of The Recovery Rate of Bell’s Palsy in Prednisolone Group and Combined Treatment Group

P-Value	Combined Group	Prednisolone group	Group
Complete	15(65.2%)	18(90%)	0.04
Partial	6(26.1%)	2(10%)	
No response	2(8.7%)	0(0%)	

## Discussion

There is no consensus on how to treat Bell’s palsy. In our study, the rate of complete recovery was 65.2% and 90% in patients who received prednisolone alone and in those who received prednisolone and acyclovir, respectively (p = 0.04). Several studies have shown higher performance and greater improvement rate of combination therapy of prednisolone and acyclovir compared with prednisolone alone (13-16). Another study also demonstrated that patients with Bell’s palsy who were treated with prednisolone and valacyclovir expressed better results and improvement than with any treatment (17). In a study by Hato et al., the recovery rate in patients treated with valacyclovir and prednisolone was higher than for patients treated with prednisolone alone. This outcome is consistent with the results of our study (18). Rosenblum R indicated better results were obtained when adding antiviral agents to corticosteroids for treating Bell’s palsy (19). Moreover, a combination of prednisolone and famciclovir was more effective than prednisolone alone in the treatment of Bell’s palsy and a significant number of patients improved by adding famciclovir (20). Adding an antivirus to the treatment of Bell’s palsy is because of HSV involvement in facial nerve inflammation. In fact, antiviruses eradicate the virus while corticosteroid reduced nerve swelling (21). However, other studies that underestimate the efficacy of treatment by acyclovir as follows. In a double blind, placebo-controlled, randomized study, early treatment with prednisolone significantly improved Bell’s palsy. However, no significant advantage was found for acyclovir alone or in combination with prednisolone (22). Kawagachi et al. showed that the reactivation of VZV, HSV-1 in 34% of patients with Bell’s palsy, and the recovery rate in patients receiving combined prednisolone and valacyclovir was significantlyy greater than prednisolone alone (23). In a meta-analysis, results of antiviral therapy were remarkably worse than for corticosteroids (n = 768, RR 2.82, 95% CI 1.09 to 7.32), while the results of antiviral and corticosteroid therapy were significantly better than a placebo (n = 658, RR 0.56, 95% CI 0.41 to 0.76) (21). Meyer showed that the effect of corticosteroids in the treatment of Bell’s palsy, despite adding antivirals, is remarkable (24). Another study concluded that steroids are effective in patients whose Bell’s palsy is started recently, and that antiviral therapy does not significantly improved the facial nerve function (25). Numthavaj et al. inferred that the recovery rate with combination therapy increases only slightly and that prednisolone is the basis of Bell’s palsy treatment (1). As maintained by the results of this study and other similar studies, combination therapy, especially in severe cases of Bell’s palsy, is more effective than prednisolone alone and results in more complete recovery of facial paralysis and reduction of its complications. However, some studies have not indicated further merits for adding antiviral agents to corticosteroids. This could be due to several factors. Also, our study was different in consumption of antiviral agents, sample size, patient age, and different inclusion and exclusion criteria in comparison of other studies. 
